# Economic Impact of COVID-19 Pandemic on Dental Practices in Germany: A Cross-Sectional Survey

**DOI:** 10.3390/ijerph19116593

**Published:** 2022-05-28

**Authors:** Thomas Gerhard Wolf, Adrian Barth, Joachim Hüttmann, Michael Lennartz, Ernst-Jürgen Otterbach, Christian Brendel, Maria Grazia Cagetti, James Deschner, Guglielmo Campus

**Affiliations:** 1 Department of Restorative, Preventive and Pediatric Dentistry, School of Dental Medicine, University of Bern, CH-3010 Bern, Switzerland; adrian.barth@students.unibe.ch (A.B.); guglielmo.campus@unibe.ch (G.C.); 2 Department of Periodontology and Operative Dentistry, University Medical Center of the Johannes Gutenberg-University Mainz, D-55131 Mainz, Germany; james.deschner@uni-mainz.de; 3 Free Association of German Dentists, Freier Verband Deutscher Zahnärzte (FVDZ), D-53117 Bonn, Germany; jhuettmann@t-online.de (J.H.); lennartz@lennmed.de (M.L.); e-j.otterbach@fvdz.de (E.-J.O.); 4 Solvi GmbH, D-65529 Waldems, Germany; c.brendel@solvi.de; 5 Department of Biomedical, Surgical and Dental Sciences, University of Milan, Via Beldiletto 1, 20142 Milan, Italy; maria.cagetti@unimi.it; 6 Department of Surgery, Microsurgery and Medicine Sciences, School of Dentistry, University of Sassari, 07100 Sassari, Italy

**Keywords:** COVID-19, corona virus, dentistry, dental practice, economic effects, global pandemic, Germany

## Abstract

An observational cross-sectional survey was planned and carried out to evaluate the economic impact of the SARS-CoV-2/COVID-19 pandemic on dental practices in Germany. An online-questionnaire was developed and previously calibrated by a group consisting of experts from dentists, lawyers, and business economists (n = 21; Intra-Class-Coefficient > 0.8). It consisted of four main categories: vital statistics, professional activity and practice structure, economic impact of the COVID-19 pandemic and validation and contextualization to avoid automated filling in. The questionnaire was administered anonymously to 9732 dentists in Germany, 4434 of whom opened it and 1496 of whom fully completed it. These results were evaluated and summarized. Respondents were divided into seven German economic macro areas. Difference in proportion among questionnaire items was evaluated with χ^2^ test or Fisher exact test appropriately. Linear trend analysis was performed among German macro areas. Ordinal multinomial linear regression analysis was run to evaluate the association with questionnaire items with respect to a collapse and/or quarantine measures due to a positive test/infection/disease of dental personnel or an increase in average monthly costs due to the pandemic. One-third experienced a collapse or quarantine measures of the predominantly self-employed participating dentists (92%). Small practices were less affected than larger ones. Average monthly costs increased sharply in all practice structures. The findings shall help to better manage future pandemics and provide information to policy makers. As the pandemic situation is still ongoing, the medium- and long-term economic impact should be further evaluated.

## 1. Introduction

The novel coronavirus SARS-CoV-2 (Severe Acute Respiratory Syndrome Coronavirus 2)-COVID-19 pandemic has caused a deep impact on the world’s health [1]. The SARS-CoV-2 human-to-human transmission has been described as being through airborne droplets or direct contact with cases or with contaminated surfaces [2]. Transmission occurs through the inhalation of respiratory droplets and/or aerosol particles, contact with fomites, and person-to-person contact [3]. Droplets are deposited in the upper airways, while smaller, aerosolized particles can penetrate lower airways and deposit in alveoli. Smaller particles can also stay airborne longer, prolonging the amount of time people might be exposed to the virus [4].

The Robert Koch Institute (RKI) Berlin (Germany) stated that, for the prevention of avoidable infections, only emergency dental treatments were to be practiced [5], leading to the assumption in April 2020 that the risk of cross-infection in dentistry might be considered considerably high [6]. Rotary dental and surgical instruments create a visible spray that can contain particle droplets of water, saliva, blood, microorganisms, and other debris. Larger droplets may lead to viral transmission to nearby subjects, whereas smaller droplets contaminated with air-suspended viral particles may provide long-distance transmission [6].

In Germany, the National Association of Statutory Health Insurance (Kassenzahnärztliche Bundesvereinigung (KZBV) and the German Federal states’ dental chamber (Bundeszahnärztekammer (BZÄK), together with the regional dental authorities (Kassenzahnärztliche Vereinigungen der Länder (KZV), have developed a joint package of measures to maintain the provision of dental care [7]. Primary dental care services were advised to radically reduce and subsequently stop routine dental care and patient face-to-face contact, and arrangements had to be made for the provision of care in urgent dental care centers during the pandemic [7].

The mandatory reduction of dental care to emergency treatment was accompanied by serious economic consequences, while at the same time the costs of maintaining the dental practice, such as staff salaries or rent, remained largely constant. Additionally, the prices for medical supplies such as protective masks (FFP2) or disinfectants, rose exponentially due to higher demand, and the economic pressure on dentists increased. The mentioned reasons for suspending dental practice in Poland during the first lockdown period in 2020 were the shortage of personal protective equipment, a general feeling of fear, insecurity, unpreparedness, and subjective perception of the COVID-19 infection risk [8]. In Switzerland, dentists reduced practice activity to a minimum of 0–10% [9]. While nearly two-thirds of dentists reported a workload reduction of more than 50% compared to the pre-pandemic period, there were enormous differences between the different German regions [10]. Weekly workload during the lockdown was reduced in over 90% of participants, in both urban and rural areas [10].

At the beginning of the SARS-CoV-2/COVID-19 pandemic, an observational cross-sectional survey was conducted to analyze the reduction in workload of German dentists during the initial lockdown in Germany [10]. However, it was not possible to estimate or predict the short-, medium-, or long-term course of the pandemic or its impact on dental practices in Germany.

The current study was then planned with the aim of measuring and analyzing the economic impact of the COVID-19 pandemic on dental practices in Germany.

## 2. Materials and Methods

### 2.1. Development and Standardization of the Questionnaire

The development of the questionnaire was partially based on the global survey of the COVIDental Collaboration Group on the effect of the pandemic on dental profession [11,12]. The original questionnaire has four domains: the first, a short introduction explaining the aims of the survey and its description and personal information of the participants; the second domain contains socio-demographic information of the participants’ professional practice and practice structure (i.e., age, sex, work status, region and area of living and details of dental practice); the third one was the main core of the questionnaire (i.e., economic consequences/impact of COVID-19 on dental practice); and the fourth domain was on the validation and contextualization of the survey to avoid automated filling in [11].

The questionnaire was standardized before the start of the survey on a small expert group of dentists, lawyers, and business economists (n = 25) and a test–retest and intra-rater reliability of each item, an Intraclass Correlation Coefficient (ICC) was run by pre-testing the questionnaire on a small group of dentists (n = 21). An ICC value of 0.80 or higher was considered satisfactory. The questionnaire was written in German, due to the national background of the participants with the origin of the German-speaking areas of Germany and partly of Switzerland.

### 2.2. Online Survey

The Ethics Committee of the Rhineland-Palatinate Medical Association (Landeszahnärztekammer Rheinland-Pfalz, Mainz, Germany) approved the questionnaire with a positive vote on 24 March 2021 (Reference Number 2021-15770). It was online and available for fourteen days (3–17 April 2021). All participants of the survey were members of the Free Association of German Dentists (Freier Verband Deutscher Zahnärzte, FVDZ, Bonn, Germany). The FVDZ is the biggest independent liberal federal association of dentists in Germany. A total of 9732 members of the FVDZ were contacted by e-mail asking for their participation. Lime Survey, a free online survey application, was used to receive und recover the data in the database of Lime Survey (Version 4.3.14, Lime Survey GmbH, Hamburg, Germany). The guidelines of the declaration of Helsinki of 1964 with its ancillary alterations served as a default for this study.

### 2.3. Data Analysis

All the data retrieved from the questionnaires were imported in a spreadsheet (ExcelTM 2021 for Mac, Microsoft, Redmond, WA, USA), then checked cleaned and finally transferred in STATA 17TM (StataCorp LLC, College Station, TX, USA) for the statistical analysis.

The dentists’ working region and area were categorized in macro areas as previously described [10]: Baden-Württemberg (BW), Bayern (BY), Nordrhein-Westfalen (NW), Berlin, Brandenburg, Mecklenburg-Vorpommern (BE-BB-MV), Bremen, Hamburg, Niedersachsen, Schleswig-Holstein (HB-HH-NI-SH), Hessen, Rheinland-Pfalz, Saarland (HE-RP-SL), Sachsen, Sachsen-Anhalt, and Thüringen (SN-ST-TH). The area of practice was categorized as rural towns (2000–5000 inhabitants), small towns (5000–20,000 inhabitants), medium towns (20,000–100,000 inhabitants) and large towns (more than 100,000 inhabitants). Participants’ work status was grouped as: employed in a private practice; owner of a private practice; private practice/state healthcare; state healthcare dentist. The type of practice was coded as: single practice; individual practice with an employed dentist; medical/dental care centers with several employed dentists under the direction of a non-dentist managing director; joint practice with several independent dentists; and university/public health. The percentage of weekly workload during the different phases of the pandemic were also recorded. Absolute and relative frequencies were calculated for each item. Difference in proportion was evaluated with χ^2^ test or Fisher exact test if one cell had a value of less than five. Multiple testing for post hoc estimation, such as the number of observed frequencies, expected frequencies, percentage, and contribution to the chi-square were run. Linear trend analysis was performed among the German macro areas’ percentage of reduction during the lockdown period of workload time assuming a 42 h normal weekly working routine. Ordinal multinomial linear regression analysis was run to estimate the association with questionnaire items with respect to a collapse and/or quarantine measures due to a positive test/infection/disease of dental personnel or an increase in average monthly costs due to the pandemic.

## 3. Results

**Figure 1:**
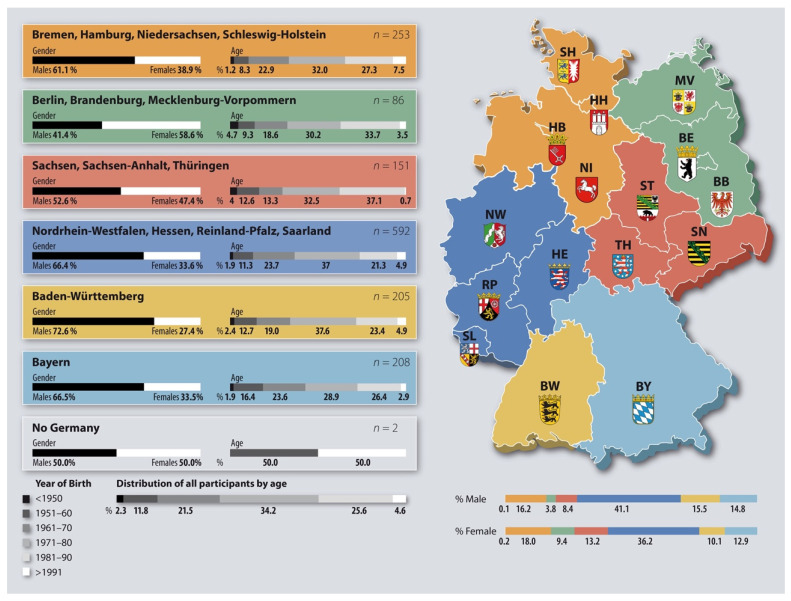
Distribution of sex, age and provenance of the participants.

Overall, the questionnaire has been administered anonymously to 9732 dentists in Germany; 4434 (45.56%) opened the link and 1496 (15.37% of the general sample) fully completed the questionnaire. The participants’ distribution by sex, age and provenance/working areas is displayed in Figure 1. Most of the participants, a total of 219, were between 40 and 50 years old and came from North Rhine-Westphalia (NW). Most of the participants (92.08 %) were self-employed, specifically from North Rhine-Westphalia (NW) (n = 550), followed by a group from Free Hanseatic City of Bremen (HB), Free Hanseatic City of Hamburg (HH), Schleswig-Holstein (SH) und Lower Saxony (NI) (n = 238) (Table 1).

Almost one third of all participants (32.40%; 32.46%; 32.46%) experienced a collapse or quarantine measures. Most of them are self-employed in an individual practice, and aged between 41 and 59 years (Table 2). Participants who have their practice in a small/medium-town, with a population between 20,000 and 100,000, make up the largest percentage of all respondents (n = 410). Out of these 410 participants from a small/medium town, most have their practices in either one of the states of North Rhine-Westphalia, Hessen, Rhineland-Pfalz, or Saarland (n = 173). More than a third of all respondents, independent of age, employment relationships and practice structure, did experience some kind of downtime and/or quarantine measures due to a positive test/infection/disease of dental personnel. However, respondents whose location of work is in North Rhine-Westphalia had the largest share (n = 175, 30.38%) of experienced downtime and/or quarantine measures. Regarding the practice structure, mainly individual practices did avoid a workload reduction due to quarantine measures (n = 522, 75.65%) because of a positive test/infection/disease of dental personnel. The ratio of quarantine measures performed (n = 15, 44.12%) and not performed (n = 19, 55.88%) was significantly more balanced in ambulatory healthcare centers with several employed dentists (Table 3).

## 4. Discussion

### 4.1. Description of the COVID-19 Situation in Germany

The current survey was conducted when the coronavirus and its effects on the human body were already better known. Since the beginning of the pandemic, numerous adjustments have been made in the management of the virus to prevent its spread. When the dentists participated in the survey (April 2021), Germany had already experienced several lockdowns as the 1st lockdown occurred from the end of March 2020 to the beginning of May 2020, a so-called “lockdown light” from the beginning of November to mid-December 2020, and the 2nd major lockdown (hard lockdown) from mid-December 2020 to mid-January 2021 with nationwide restrictions that were implemented by laws or, predominantly, ordinances by the governmental authorities. The lockdowns led to a standstill in large parts of the economy and social life as well as to drastic changes in the healthcare system. Elective procedures were postponed creating more capacity, and only dental emergencies were allowed during the 1st lockdown [5,7]; the situation in public hospitals was extremely tense.

Patient reluctance to undergo planned surgeries, necessary protective measures, and increased hygiene requirements, as well as vacant admission capacity for COVID-19 patients in intensive care units, further exacerbated the situation for hospitals, which was already precarious before the pandemic [13]. Dentists belong to so-called system-relevant professions in Germany. Dental practices and clinics had to continue operating regardless of the lockdowns for at least emergency treatments as long as there were no quarantine measures resulting in the closure of the practice after, for example, a case of a COVID-19 infection of a dentist or the staff. However, weekly workload was drastically reduced in over 90% of participating dentists during the initial lockdown in Germany; nearly two-thirds of dentists reported a reduction in their workload of more than 50% compared to the period before COVID-19 [10].

### 4.2. Purpose of the Study and Main Results

The aim of the current study was to measure and analyze the economic impact of the COVID-19 pandemic on dental practices in Germany. Most of the participating dentists were self-employed and between 41 and 59 years old. Individual dental practices were less affected by the economic impact of COVID-19 compared to larger practice structures, with personnel expenses being the largest cost item at 40% when considering the business cost structure of an average dental practice [14]. Approximately one-third of all participants experienced an outage or quarantine measures. There was an increase in the average monthly cost for practice structures due to the COVID-19 pandemic, and significant differences were observed with respect to place of practice.

### 4.3. Economic Impact of COVID-19 on Dental Practice Related to Study Results

Profound economic effects on dental practices in Germany, using a mathematical theory of probability—the Markov- and the Monte-Carlo-model—could be observed due to COVID-19 and associated policies at the beginning of the pandemic [15], confirming the results of the current study. A lockdown with accompanying mitigation/suppression led to a decline in the utilization of all services, mainly in preventive services, periodontic and prosthodontic services of more than 70% each; accompanied by mean revenue losses of approximately 15–19% for both public and private insurance as well as additional payments for special treatments [15]. The income of Iraqi dentists decreased by about half for 75% of participating patients, independently of demographic variables [16], confirming the results for Germany [10], Switzerland [9], and Poland [8].

A price increase of more than 75 was reported by nearly 30% of respondents, according to the survey, while at the same time the number of patients had also decreased by up to half. While there were government support programs for dentists, most dentists were not eligible to apply for them [16]. We hypothesized that the different economic impact of the pandemic on large dental practices compared to single-professional practices could be due to the costs that large facilities faced both during the lockdown and at the reopening. Moreover, a single-professional structure can respond to the different needs and restrictions that occurred during all phases of the pandemic.

This has parallels with the situation in Germany, where government support could also be declared as a failure for the practice owner in the case of quarantine imposed by the local health authority organized by the Ministry of Health of each country (Bundesland). However, it contrasts with the situation in Switzerland, where dentists were not paid any support from the state in the event of practice failure due to the COVID-19 pandemic when the practice owner was quarantined.

The timing of conducting the survey plays an important role in the rapidly changing pandemic situation. While the Iraqi dentists’ survey data in July 2020 refer to the time since the outbreak of the pandemic, the present study targets the entire year of 2020 and was conducted in April 2021. It is remarkable in both studies that enormous economic impacts could be observed and yet the number of practice staff has remained largely constant and has not been reduced [16].

Generalizations of the data or economic impact on dental practices cannot be made due to local, regional, and national differences in terms of the COVID-19 pandemic.

### 4.4. Limitations

Various limitations must be considered. It is an anonymous questionnaire, so answering the questions could be completed multiple times by one participant. Additionally, the short period of data collection must be mentioned, even though the situation was not an immediately changing situation as in the previous study [10], but a closed period of the year 2020. The number of participating dentists is relatively small in relation to the whole dental profession in Germany with a total of 72,468 practicing dentists [14]. In survey studies, the participation rate is an important sign of the representativeness of the results. Participation rates have been declining in all kinds of surveys during the past decades [17,18]. The response rate that every researcher should pursue is 100%. Few researchers enjoy such a high figure. Even though the questionnaire was opened by more than 50% of the participating dentists in this study, only one third of them answered it completely. Based on the experience from previous studies [9,10,11,12,13,14,18,19], the authors, together with the expert group, limited the number of questions that were considered most relevant. The aim was to obtain the best possible results so that the information obtained would help dentists and policy makers to make better decisions for this and future pandemics. Accordingly, an actual verification of the data with actual data on billing or the economic circumstances of the respective dental practice structure could provide additional information in a further study.

## 5. Conclusions

This study examines the economic impact of the COVID-19 pandemic. It could be observed that:

1. One-third of the predominantly self-employed participating dentists experienced a collapse or quarantine measures;
2. Interruption of dental practice operations due to an obliged quarantine or collapse results in a significant loss of revenue and a tremendous increase in cost;
3. There were geographic variations in practice closures, which could be dependent on the number of practice employees;
4. Small dental practices were less affected than larger practice structures;
5. Average monthly costs increased sharply across all practice structures.

In summary, this survey provides a closer look at the resulting situation for practicing dentists in Germany, what economic effects they experienced in the course of the pan-demic and what reasons may be causal. It can be stated that, a discontinued dental practice working shift due to a prescribed quarantine can especially induce significant losses in the generation of revenue. Assuming these circumstances, this paper discusses that the proportion of actual practice closures varies geographically as well as depends on the number of employees of the practice with both physician and non-physician staff. The findings shall help better manage future pandemics and provide information for policy makers. As the pandemic is ongoing, the medium- and long-term economic impact should be further investigated.

## Figures and Tables

**Table 1:** Distribution of the participants.

**(a)** Working areas (expressed as German macro areas) and number of inhabitants of the work location.						
Macro Area	Village/Rural (<2000–≤5000) n (%)	Small Town (>5000–≤20,000) n (%)	Small/Medium-town (>20,000–≤100,000) n (%)	Small City (>100,000–≤500,000) n (%)	Large City (>500,000) n (%)	Total n (%)
HB-HH-SH-NI (Berlin, Brandenburg, Mecklenburg-Vorpommern)	42 (16.41)	69 (26.95)	69 (26.95)	28 (10.94)	48 (18.75)	256 (16.87)
BE-BB-MV (Berlin, Brandenburg, Mecklenburg-Vorpommern)	9 (10.23)	12 (13.64)	17 (19.32)	5 (5.68)	45 (51.14)	88 (5.80)
SN-ST-TH (Sachsen, Sachsen-Anhalt, Thüringen)	35 (22.73)	36 (23.38)	39 (25.32)	27 (17.53)	17 (11.04)	154 (10.15)
NW-HE-RP-SL (Nordrhein-Westfalen, Hessen, Rheinland-Pfalz, Saarland)	56 (9.33)	129 (21.50)	173 (28.83)	149 (24.83)	93 (15.50)	600 (39.55)
BW (Baden-Württemberg)	29 (13.94)	60 (28.85)	69 (33.17)	35 (16.83)	15 (7.21)	208 (13.71)
BY (Bayern)	44 (20.85)	66 (31.28)	42 (19.91)	25 (11.85)	34 (16.11)	211 (13.91)
Total	215 (14.15)	373 (24.56)	410 (26.99)	269 (17.71)	252 (16.59)	1517
Subjects not working in Germany n = 2 (0.13%) c2(19) = 164.27 *p* < 0.01.						
**(b)** Working areas (expressed as German macro areas) and employment type.						
Macro Area	Self-Employed n (%)		Salaried n (%)		Total n (%)	
HB-HH-SH-NI (Berlin, Brandenburg, Mecklenburg-Vorpommern)	238 (93.33)		17 (6.67)		255 (16.83)	
BE-BB-MV (Berlin, Brandenburg, Mecklenburg-Vorpommern)	81 (92.05)		7 (7.95)		88 (10.65)	
SN-ST-TH (Sachsen, Sachsen-Anhalt, Thüringen)	141 (91.56)		13 (8.44)		154 (39.34)	
NW-HE-RP-SL (Nordrhein-Westfalen, Hessen, Rheinland-Pfalz, Saarland)	550 (92.13)		46 (7.71)		596 (13.73)	
BW (Baden-Württemberg)	192 (92.75)		16 (7.25)		208 (13.73)	
BY (Bayern)	193 (91.00)		19 (9.00)		212 (13.99)	
Total	1395 (92.08)		120 (7.92)		1515	
Subjects not working in Germany n = 2 (0.13%); not-responders n = 2 (0.13%) c2(11) = 26.05 *p* = 0.01.						

**Table 2:** Survey of the participants on whether they experienced a collapse and/or quarantine measures due to a positive test/infection/disease of dental personnel. The participants were stratified by age, employment relationships and which practice structure they work in.

Did your practice experience a collapse and/or quarantine measures due to a positive test/infection/disease of dental personnel?			
	NO n (%)	YES n (%)	Total n (%)
≤40 years	124 (61.08)	79 (38.92)	203 (14.24)
41–59 years	530 (66.25)	270 (33.75)	800 (56.10)
≥59 years	310 (73.29)	113 (26.71)	423 (29.66)
Total	964 (63.46)	462 (36.54)	1426
Not responders n = 93 (6.12%) χ^2^ = 10.85, *p* < 0.01			
Practice of dentistry			
Individual practice with one salaried dentist	183 (60.00)	122 (40.00)	305 (21.06)
Practice with several employed dentists under the management of one dentist	57 (55.88)	45 (44.12)	102 (7.04)
Individual practice	522 (75.65)	168 (24.35)	690 (47.65)
Joint practice (several practice owners)	196 (61.64)	122 (38.36)	318 (21.96)
Ambulatory healthcare center with several employed dentists under the direction of a dentist or a managing director (non-dentist)	20 (60.61)	13 (39.39)	33 (2.28)
Total	978 (67.54)	470 (32.46)	1448
Not responders n = 71 (4.67%) χ^2^ = 40.73, *p* < 0.01			

**Table 3:** Survey of the participants if they experience a collapse and/or quarantine measures due to a positive test/infection/disease of dental personnel. The respondents were stratified by sex, age, macro area, practice structure and which specialization the practice in which they work has.

Did your practice experience a collapse and/or quarantine measures due to a positive test/infection/disease of dental personnel?			
Logistic regression; number of observations = 1411, log-likelihood = −880.24 χ^2^ = 16.71, *p* = 0.01			
	Odds Ratio (SE)	*p*-value	95% Confidence Intervals
Sex	1.04 (0.12)	0.75	0.82/1.31
Main place of practice	0.97 (0.05)	0.57	0.88/1.07
Age category	0.77 (0.07)	0.00	0.64/0.92
Macro area	1.37 (0.31)	0.16	0.88/2.13
Practice structure	0.92 (0.05)	0.09	0.83/1:01
Specialization	0.72 (0.27)	0.38	0.34/1.50
Did your practice experience an increase in average monthly costs due to the pandemic?			
Logistic regression; number of observations = 1434, log-likelihood = −102.38 χ^2^ = 15.71, *p* = 0.01			
	Odds Ratio (SE)	*p*-value	95% Confidence Intervals
Sex	1.07 (0.51)	0.89	0.42/2.74
Main place of practice	0.61 (0.15)	0.04	0.39/0.98
Age category	1.33 (0.48)	0.42	0.66/2.72
Macro area	1.31 (0.15)	0.67	0.80/2.74
Practice structure	1.01 (0.20)	0.92	0.68/1.50

## Data Availability

The data are not publicly available due to the General European Data Protection Regulation (GDPR) of 25 May 2018.The data presented in this study are available on request from the corresponding author.
